# Curcumin supplementation and delayed onset muscle soreness (DOMS): effects, mechanisms, and practical considerations

**DOI:** 10.20463/pan.2020.0020

**Published:** 2020-09-30

**Authors:** Wan-Young Yoon, Kihyuk Lee, Jooyoung Kim

**Affiliations:** 1 Department of Health Care Exercise, Seowon University, Cheongju, Republic of Korea; 2 Department of Sport Culture, Dongguk University, Seoul, Republic of Korea; 3 Office of Academic Affairs, Konkuk University, Chungju, Republic of Korea

**Keywords:** curcumin, delayed-onset muscle soreness, eccentric exercise, inflammatory response, muscle damage, recovery

## Abstract

**[Purpose]:**

In this literature review we aimed to investigate the effects of curcumin supplementation on delayed onset muscle soreness (DOMS), which occurs after exercise, and evaluate related parameters to propose practical recommendations for the field of exercise physiology.

**[Methods]:**

Experimental studies conducted on curcumin supplementation and DOMS were systematically reviewed to determine (1) the effect of curcumin supplementation on DOMS, (2) potential mechanisms by which curcumin supplementation may attenuate DOMS, and (3) practical considerations for curcumin supplementation.

**[Results]:**

While several studies have reported that curcumin supplementation attenuates DOMS after exercise, others have reported that curcumin supplementation has no effect on DOMS. Several mechanisms have been proposed by which curcumin supplementation may attenuate DOMS; the most probable of which is a reduction in inflammatory response. Other potential mechanisms include modulation of transient receptor potential vanilloid 1 (TRPV1) or changes in post-exercise capillary lactate levels; these require further examination. The usual recommended dose of curcumin is 150–1500 mg daily (sometimes up to 5 g), divided into 2–3 portions and taken before and after exercise. It is not necessary to take curcumin together with piperine.

**[Conclusion]:**

Although conflicting results regarding the effects of curcumin supplementation on DOMS exist in literature, it may be considered as a method of nutritional intervention for reducing post-exercise DOMS.

## INTRODUCTION

It is widely accepted that regular exercise promotes health and improves physical fitness. However, repeatedly performing high-intensity exercise that involves unaccustomed or eccentric muscle contractions can lead to delayed-onset muscle soreness (DOMS) [1,2]. Researchers have proposed several hypotheses for the etiology of DOMS previously such as lactic acid, muscle spasms, muscle damage, and inflammatory response, whereas novel hypotheses include Toll-like receptor 4 activation, increased levels of neurotrophic factors such as nerve growth factors and glial cell line–derived neurotrophic factors; however, the exact cause of DOMS remains unclear [3–7].

DOMS typically appears within 24 hours after exercise, peaks within 24–72 hours, and fully subsides after 5–7 days [8]. DOMS prolongs recovery time; hence, an individual with DOMS may not be able to follow their exercise routine and may be psychologically affected negatively. DOMS can also reduce performance or training quality in athletes [9,10]. Exercise physiologists have extensively investigated various methods to attenuate post-exercise DOMS [11,12]. Nutritional intervention is one such method that has been widely used to attenuate or prevent DOMS among both athletes and the general population [13,14].

Curcumin, also called diferuloylmethane, is a well-known spice used in curry in India and other Asian countries. It is a natural polyphenol that has attracted attention for its anti-inflammatory, antioxidant, and anticancer activities [15,16]. Studies have found that curcumin significantly reduces pain from burns as well as pathological pain caused by sciatic nerve injury, spinal cord injury, diabetic neuropathy, and alcoholic neuropathy [16,17]. Researchers in the field of exercise physiology have investigated the effect of curcumin on post-exercise DOMS, with a few studies reporting that curcumin supplementation can effectively attenuate DOMS after high-intensity eccentric exercise [18-20].

Although these reports suggest that curcumin supplementation can be an effective and helpful nutritional intervention for individuals suffering from DOMS after exercise, there are only a few comprehensive reviews of the literature on this topic. Therefore, we reviewed experimental studies on the effect of curcumin supplementation on DOMS and related mechanisms, with the aim of proposing practical recommendations for the field of exercise physiology.

### Effect of curcumin supplementation on DOMS

Attenuating DOMS after exercise is important for recovery, as it allows the individual to quickly move on to the next exercise with maintained or improved intensity; thus, increasing the likelihood of achieving the performance target [21]. For this reason, several studies have investigated the effect of curcumin on post-exercise DOMS, using various exercise protocols [18-20,22].

Several studies have reported that taking curcumin supplements before and after exercise attenuates DOMS. Nicol et al. [19] instructed 17 men to take 5 g of curcumin (2.5 g per dose, two doses per day) starting 2 days before exercise and ending 3 days after exercise. They reported that taking curcumin within 24–48 hours after eccentric singleleg press exercise reduced DOMS in the lower limbs during single-leg squat, gluteal stretch, and squat jump exercises. In addition to reducing DOMS, the supplement also increased single-leg jump performance, suggesting that DOMS attenuation positively affects performance and promotes recovery. Mallard et al. [18] instructed 28 men to take 450 mg of curcumin before and after performing resistance exercise of the lower limbs to the point of exhaustion; they reported that the curcumin supplementation group had significantly lower levels of DOMS than the placebo group at 48 and 72 h after exercise. In a study of 20 men, Drobnic et al. [23] reported that compared to that of the placebo, administration of curcumin twice a day (200 mg per dose, morning and evening, starting 2 days before exercise and ending 24 h after exercise) significantly reduced DOMS in the lower limbs following downhill running.

Other studies have examined the effects of single-dose curcumin supplementation before or after exercise on DOMS. Amalraj et al. [24] reported that single-dose supplementation with 500 mg of curcumin 1 h before exercise significantly reduced DOMS after downhill running. Nakhostin-Roohi et al. [25] instructed 10 men to perform repeated quadriceps muscle contractions using a squat machine, inducing muscle damage, and found that those who took a single 150-mg dose of curcumin immediately after exercise had significantly reduced DOMS 48 and 72 h later.

Tanabe et al. [20] performed two experiments in which 20 men were instructed to perform eccentric exercise of the elbow flexors using an isokinetic dynamometer. In the first experiment, the participants took curcumin supplements before exercise for 7 days. In the second experiment, the participants took curcumin supplements after exercise for 7 days. In both experiments, the participants’ daily curcumin intake was 180 mg (90 mg per dose). While curcumin supplementation before exercise produced no significant effect on DOMS compared to that of the placebo, curcumin supplementation after exercise significantly reduced DOMS after 3–6 days. The authors reported similar results in their subsequent study [26], in which they instructed 24 men to perform eccentric exercise as in the previous study, with some participants taking 180 mg of curcumin (90 mg per dose) for 7 days before exercise and others taking the same dose for 4 days after exercise. Once again, curcumin supplementation after exercise was found to effectively reduce DOMS, whereas curcumin supplementation before exercise did not affect DOMS or any other parameters associated with muscle damage.

One recent study examined the effect of long-term curcumin supplementation on DOMS. Basham et al. [22] investigated the effect of daily intake of 1.5 g of curcumin (three 500 mg capsules) for 28 days in 19 men with muscle damage induced by aerobic step bench exercise and found that curcumin supplementation reduced DOMS more significantly than the placebo.

However, some studies have reported that curcumin supplementation has little or no effect on DOMS [27-31]. Cardaci et al. [27] instructed 23 men and women to take 2 g of curcumin for 7 days before engaging in downhill running and 4 days afterward; they reported no significant difference compared to the placebo group with regard to the effect on DOMS during the recovery phase. Delecroix et al. [28] reported that 17 elite male rugby athletes took 6 g of curcumin (2 g per dose, 3 doses per day) 48 h before a repeated single-leg jump exercise on a downhill slope and 48 h after the exercise; both the curcumin supplementation and placebo groups reported an increase in DOMS after exercise, with no significant difference. In a study by McFarlin et al. [29], 28 men and women took 400 mg of curcumin per day, 2 days before muscle damage induction by leg press exercise and 3 days afterward, there was no significant difference between the curcumin supplementation and placebo groups with regard to DOMS in the quadriceps or DOMS during activities of daily living.

In another study by Tanabe et al. [30], 14 men took 150 mg of curcumin both 1 h before eccentric exercise of the elbow flexors and 12 h after the exercise, and muscle damage parameters were measured. While curcumin supplementation mitigated the post-exercise increase in the level of creatinine kinase, which is a marker of muscle loss and muscle membrane disruption, it did not have any effect on DOMS. In a study by Jäger et al. [31], 63 physically active men and women were administered a placebo, 50 mg of curcumin, or 200 mg of curcumin every day for 8 weeks (approximately 56 days); they then performed downhill running in order to induce muscle damage. DOMS was found to be more effectively reduced in the subjects who took 200 mg of curcumin compared to that of those who took 50 mg, but the difference was not statistically significant.

Possible reasons for these conflicting results include differences in the characteristics of the participants, methods of curcumin supplementation, and exercise models and protocols used. The study by Delecroix et al. [28] included elite rugby athletes rather than individuals from the general population. In the studies by Cardaci et al. [27] and Delecroix et al. [28], curcumin was administered together with 20 mg of piperine, which is a major constituent of black pepper. In their most recent study, Tanabe et al. [30] used a curcumin dose smaller than the doses used in their previous studies [20,26], and Jäger et al. [31] had their participants take curcumin for a prolonged period of 8 weeks.

### Potential mechanisms for the effects of curcumin on DOMS

In general, inflammatory responses are increased by exercise-induced muscle damage [32] and are closely associated with the development of DOMS [33]. DOMS induced by inflammatory responses is accompanied by various biochemical phenomena. Phospholipase A2 promotes metabolic conversion of the phospholipids constituting the cell membranes of muscle cells to arachidonic acid, which is further converted to prostaglandins and leukotrienes, triggering an increase in mast cell numbers and histamine levels [3]. At the same time, neutrophils and macrophages promote phagocytosis in damaged muscles [11]. Substrates whose levels have been elevated through this process stimulate afferent nerve fibers connected to muscles, and the individual perceives DOMS when the stimulus reaches the medulla and cerebral cortex of the brain through the spinal cord [3].

The most probable mechanism by which curcumin supplementation may attenuate DOMS is by reducing the inflammatory responses that occur during the recovery phase after exercise. Curcumin inactivates nuclear factor kappa B (NF-κB), which is a key mediator of inflammation, leading to reduction in the levels of other inflammatory mediators including pro-inflammatory cytokine mRNA and proteins [34]. Furthermore, curcumin can reduce the expression of the inflammatory markers intercellular adhesion molecule 1 (ICAM-1) and vascular cell adhesion molecule 1 (VCAM1). It attenuates the activity of 5-lipoxygenase (5-LOX) and cyclooxygenase-2 (COX-2), which are enzymes in the leukotriene-producing metabolic pathway [35]. Several studies on DOMS attenuation by curcumin supplementation support the hypothesis that curcumin exerts its effect through its anti-inflammatory properties [18,23,24].

A few studies have reported that curcumin supplementation has an effect on the inflammatory response that occurs after exercise-induced muscle damage [20,29,36]. Davis et al. [36] reported that curcumin supplementation significantly reduced the levels of interleukin (IL) 1-beta (IL-1β), IL-6, and tumor necrosis factor alpha (TNF-α), which are cytokines promoting inflammatory responses after downhill running. McFarlin et al. [29] reported that curcumin supplementation was associated with reductions in the levels of TNF-α and IL-8 following leg pressing. Tanabe et al. [20] reported that curcumin supplementation significantly reduced IL-8 levels 12 h after eccentric exercise. Mallard et al. [18] reported that curcumin supplementation was associated with decreased thigh circumference after exercise and increased the levels of IL-6 and IL-10, which are anti-inflammatory cytokines (IL-6 has both pro- and anti-inflammatory characteristics). Thigh circumference is an indirect marker of inflammatory response, and an increased thigh circumference is associated with DOMS [37].

However, some studies found no association between the curcumin-induced changes in inflammatory responses and DOMS [22,29]. It has been suggested that curcumin promotes the modulation of transient receptor potential vanilloid 1 (TRPV1) rather than changing the inflammatory responses to exert anti-nociceptive effects [22,26,38,39]. However, TRPV1 modulation is only one of several potential mechanisms for DOMS attenuation by curcumin supplementation, and no study has directly investigated the relationship between DOMS and TRPV1 modulation by curcumin in a model of exercise-induced muscle damage. Further research is needed to confirm this hypothesis.

Mallard et al. [19] proposed another potential mechanism by which curcumin reduces DOMS, relating it to post-exercise changes in the level of capillary lactate. Changes in lactate levels indicate changes in the muscle pH. According to Mallard et al. [18], acidosis prevention by muscle pH reduction may be a possible mechanism of DOMS reduction. However, this hypothesis has been considered for some time, and several studies have demonstrated that there is no association between lactate and DOMS and that post-exercise changes in lactate levels are not synchronous with the onset of DOMS [40,41]. Therefore, the hypothesis proposed by Mallard et al. [18], which associates changes in lactate levels with DOMS, seems improbable.

### Practical considerations for curcumin supplementation

Individuals who are new to exercising, resuming exercise, or looking for gradual improvements in their exercise programs may consider taking curcumin supplements. Of all the studies considered in this review, only one study involved trained athletes [28], and this study found that curcumin supplements did not significantly affect DOMS. Most studies included individuals drawn from the general population. The differences in the study results may be because the general population lacks exercise experience compared to athletes, and thus, be more sensitive to DOMS [42]. Further research is necessary on the effect of curcumin supplementation on DOMS in athletes.

curcumin was usually 150–1500 mg and sometimes as high as 5 g, with the supplement being administered as a single dose or over 2–3 doses [18-20,23-26]. Although more research is needed to determine which curcumin regimen most effectively reduces DOMS, multiple smaller doses of curcumin may be considered in cases where the daily intake is high. For example, if an individual needed to consume 1500 mg of curcumin every day, the amount may be split into three 500-mg doses taken in the morning, early afternoon, and evening [22]. There have been no reports of toxicity or side effects from the doses of curcumin used in these studies [43].

Based on the findings of this review, it is recommended that curcumin supplementation be performed before and after exercise for up to 3–4 days after exercise [18,19,23,26]. Although a single dose of curcumin may be taken before or after exercise [24,25], more research is needed on the effectiveness of such a method of supplementation. Tanabe et al. reported that consuming curcumin after exercise reduced DOMS more effectively than does taking curcumin before exercise [20,26]; however, these studies are the only two studies that have made this comparison. Therefore, further research should be conducted on the optimal timing of curcumin supplementation, using different exercise methods and intensities to induce muscle damage or DOMS and using a wider variety of subjects. Lastly, while curcumin may be supplemented with piperine to improve its low bioavailability and absorption [27,28], it is unclear whether this method of supplementation improves DOMS attenuation. Based on study findings till date, curcumin can sufficiently reduce post-exercise DOMS even when taken without piperine.

## CONCLUSION

In conclusion, although there are conflicting results regarding the effect of curcumin supplementation on DOMS, individuals who are new to exercising, resuming exercise, or looking for gradual improvements in their exercise programs may consider curcumin supplementation as a method of nutritional intervention to reduce post-exercise DOMS. Future studies should aim to reveal the mechanisms potentially affecting the attenuation of DOMS by curcumin more clearly, the optimal amount, and period of curcumin intake.
